# Efficacy of pre-operative stereotactic radiosurgery followed by surgical resection and correlative radiobiological analysis for patients with 1–4 brain metastases: study protocol for a phase II trial

**DOI:** 10.1186/s13014-018-1178-8

**Published:** 2018-12-20

**Authors:** Wei X. Huff, Namita Agrawal, Scott Shapiro, James Miller, Charles Kulwin, Mitesh Shah, Jesse J. Savage, Troy Payner, Alexander Vortmeyer, Gordon Watson, Mahua Dey

**Affiliations:** 1 Department of Neurosurgery, Indiana University School of Medicine, Indiana University Purdue University Indianapolis, Neuroscience Research Building, 320 W 15th Street, NB 400A, Indiana, IN 46202 USA; 2 Department of Radiation Oncology, Indiana University School of Medicine, Indiana University Purdue University Indianapolis, Indiana, USA; 3 Department of Pathology, Indiana University School of Medicine, Indiana University Purdue University Indianapolis, Indiana, USA

**Keywords:** Stereotactic radiosurgery, Brain metastases, Metastases surgery, Immune profiling, Molecular profiling

## Abstract

**Background:**

Stereotactic radiosurgery (SRS) has emerged as a common adjuvant modality used with surgery for resectable brain metastases (BMs). However, the optimal sequence of the multi-modality therapy has not been established. The goal of the study is to evaluate 6-month local control utilizing pre-operative SRS followed by surgical resection for patients with 1–4 brain metastases.

**Methods:**

This prospective, single arm, phase II trial will recruit patients with up to 4 brain metastases and at least one resectable lesion. All lesions will be treated with SRS and symptomatic lesions will be resected within 1–4 days after SRS. Patients will be monitored for 6-month local control, in-brain progression free survival, distant in-brain failure, rate of leptomeningeal spread, radiation necrosis and overall survival. Additionally, we will also perform correlative radiobiological molecular studies to assess the effect of radiation dosing on the tumor tissue and clinical outcomes. We expect that pre-operative SRS to the gross tumor prior to surgical resection will improve local control and decrease leptomeningeal failure.

**Discussion:**

Our study is the second prospective trial to investigate the efficacy of pre-operative SRS in the treatment of multiple BMs. In addition, the correlative molecular studies will be the first to investigate early response of BMs at a cellular and genetic level in response to radiation doses and potentially provide molecular prognostic markers for local control and overall survival.

**Trial registration:**

Clinicaltrials.gov identifier: NCT03398694 (registration date: January 12, 2018).

## Issue Section

Research – Human – Study protocols

## GENERAL INFORMATION

### Pre-operative stereotactic radiosurgery followed by resection for patients with brain metastases

**Study Dates**

January 12, 2018, currently recruiting

**Investigators:** Table 1

- Research Site: IU Health Methodist Hospital
  1701 N Senate Ave. Indianapolis IN 46202
  Phone # 317 962-2000

**Fig. 1:**
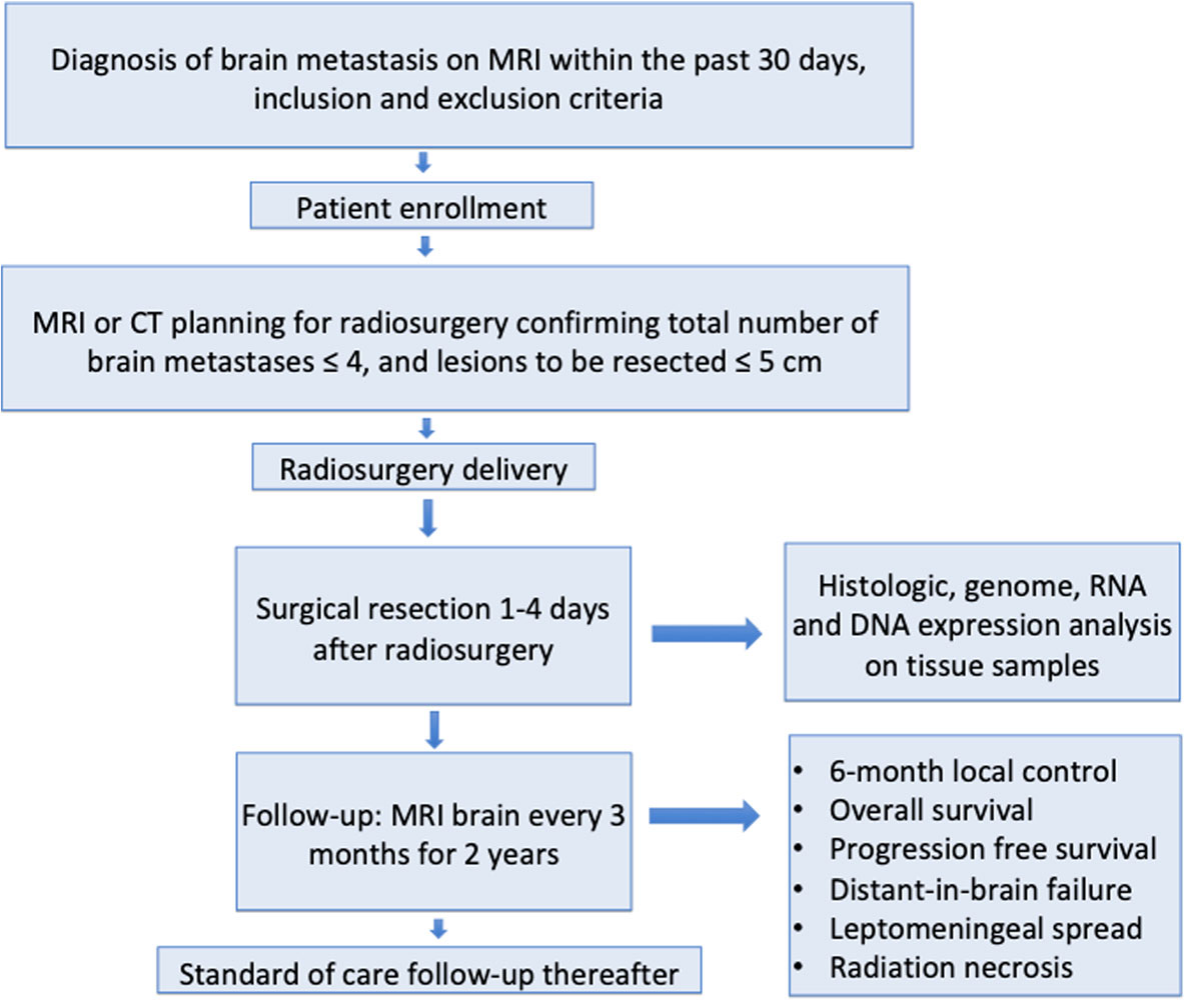
Flow chart describing the clinical trial study design

**Table 1:** Investigators Details

Name	Title	Role	Address
Gordon A. Watson MD, PhD	Associate Professor of Clinical Radiation Oncology	Principle Investigator (Clinical Trial)	535 Barnhill Drive RT 041 RAON Indianapolis IN 46202 Phone # 317–962-3172
Mahua Dey MD	Assistant Professor of Neurological Surgery Adjunct Assistant Professor of Microbiology and Immunology	Co-Investigator (Clinical Trial) Principle Investigator (Correlative Radiobiological Analysis)	Dey Laboratory Indiana University School of Medicine Neuroscience Research Building 320 W 15th Street NB 400A Indianapolis IN 46202 Phone # 317–274-2601
Alexander O Vortmeyer MD, PhD	Professor of Clinical Pathology and Laboratory Medicine	Co-Investigator (Clinical Trial and Correlative Radiobiological Analysis)	Pathology and Laboratory Med IU Health Pathology Laboratory 350 W 11th Street Room 4034 Indianapolis IN 46202 Phone # 317–274-1618
Namita Agarwal MD	Resident Physician of Radiation Oncology	Co-Investigator (Clinical Trial)	535 Barnhill Drive RT 041 RAON Indianapolis IN 46202 Phone # 317–962-3172
Sandra Althouse MS	Department of Radiation Oncology	Statistician	535 Barnhill Drive RT 041 RAON Indianapolis IN 46202 Phone # 317–962-3172

## Background

Incidence of brain metastases (BMs) is increasing with improved systemic therapies, many of which have a limited impact on intracranial disease. [1, 2] Historically a combination of surgical removal with subsequent whole brain radiation therapy (WBRT) was the gold standard for managing patients with a single, large BM and WBRT alone for patients with multiple metastases. [3, 4] Recent advances in the systemic treatment of various cancers have resulted in long-term survivors. [1, 2, 5] WBRT is associated with debilitating neurocognitive dysfunction, which results in significant impairment in the quality of life of long-term survivors. [6–9] As a result, current practice patterns shifted away from use of WBRT for oligo-metastasis, defined as limited number of BMs, reserving WBRT as salvage therapy or for disseminated BMs. [9, 10] Stereotactic radiosurgery (SRS) delivers high dose of radiation in a highly conformal way to relatively small target (< 3 cm) with minimal radiation to the surrounding normal tissue. [11] Since its conception, SRS emerged as a leading modality to treat BMs in variety of clinical scenarios including a boost with WBRT, as definitive treatment alone for patients with limited number of BMs and as an adjuvant treatment modality in pre- or post-operative setting. [12–14] Many tumors, regardless of whether they are radiosensitive or resistant, single or multiple, can be adequately managed with SRS. [10, 11, 15]

Although there has been no consensus on combination therapy with surgery and SRS [16, 17], surgical resection continues to play an important role in patients with a limited number of BMs and relatively good performance status where: 1) pathological diagnosis is needed, 2) a large BM (> 2 cm) causing significant mass effect necessitating direct decompression, and/or 3) neurological signs and symptoms refractory to corticosteroid management. [18, 19] Therefore, for patients with 1–4 BMs, especially in the above mentioned scenarios, adjuvant radiosurgery for surgically resected lesions is a common practice to achieve high LC. [9, 10]

Several clinical studies evaluating the role of post-operative SRS in management of BMs concluded that SRS is a safe and effective adjuvant treatment strategy for BMs (Table 2). A systematic review of 14 studies involving 629 patients treated with SRS after surgical resection showed pooled LC rate of 83%, distant intracranial failure of 49% and need for salvage WBRT in 29% of cases. [20] In addition, time to salvage therapy from initial resection was found to be 8.4 months and 10–33% of patients suffered cavity-SRS induced complications such as radiation-induced edema in 43% and radiation necrosis (RN) in 23%. [20] Two recently published phase III trials of post-operative SRS by Mahajan et al. and Brown et al. demonstrated local control efficacy and neurocognitive preservation compared with WBRT, respectively. [9, 21] Mahajan et al. prospectively randomized 132 patients in a single institution phase III study comparing observation and post-operative SRS and showed a significant improvement in local tumor recurrence-free rate at 12-month in post-operative SRS arm (72%) compared to observation arm (43%). [21] In the same journal issue, Brown et al. reported a multi-center prospective randomized phase III study to compare the post-operative SRS and WBRT in a total of 194 patients and observed similar overall survival but significantly improved neurocognition in post-operative SRS compared with WBRT. [9] Interestingly, their secondary endpoint analysis demonstrated a lower surgical bed and local control rate as compared to data previously reported. [9, 21] With a 6-month surgical bed control of 80.4% and an estimated 60.5% at 12-month, the author discussed that lower LC rate after SRS could be due to falsely elevated frequency of recurrence by including “pseudoprogression” on imaging and patients and tumor mark-up differences between trials. [9] Although there has been some discrepancy in the literature regarding to local control rates in patients receiving post-operative SRS (Table 2), these studies nevertheless support the combination use of surgery and SRS as an effective way to treat brain oligometastasis to delay WBRT and the associated neurocognitive and quality of life decline.

**Table 2:** Published studies of Post-op SRS after Metastases Resection

Study	Treatment Modalities	Number of Patients	Median survival (months)	LR (%)	DBF (%)
Bahl, 2006 [36]	Op + SRS/fSRT	7	8.9	57.1	14.3
Kim, 2006 [37]	Op + GKRS	79	16	5.1	NA
Soltys, 2008 [22]	Op + CK	72	15.1	21	49.2
Iwai, 2008 [38]	Op + GKRS	21	20	23.8	47.6
Mathieu, 2008 [39]	Op + GKRS	40	13	27	54.1
Limbrick, 2009 [40]	Op + GKRS	15	20	20	60
Jagannathan, 2009 [41]	Op + GKRS	47	11	6.4	72.3
Robbins, 2012 [42]	Op + SRS	85	12.1	18.8	55.3
Johnson, 2016 [43]	Op + GKRS	112	12.9	15.6	NA
Brown, 2017 [9]	Op + SRS	98	12.2	19.6 (6 m)	NA
Mahajan, 2017 [21]	Op + SRS	63	17	28	58

The drawbacks for post-operative SRS include the need for cavity margin expansion, the unpredictability of patients’ postoperative course and potential delay in SRS treatment after surgery. Soltys et al. analyzed 76 post-operative SRS cases and demonstrated that increasing conformality index significantly correlated with improved LC and concluded that a 2-mm margin should be used around the resection cavity due to the possible imprecise nature of defining the target volume in a post-surgical setting. [22] Therefore, clinicians began to investigate an alternative paradigm of using preoperative SRS to reach excellent local control and preserve cognitive function.

Atalar et al. retrospectively analyzed the risk of leptomeningeal disease (LMD) in 175 patients with BMs treated with post-operative SRS and found that 13% developed LMD 5 months following SRS. [23] In Mahajan’s study, the LMD reached 28% at 12 month in post-operative SRS arm as compared to 16% in observation arm, though the difference did not reach statistical significance. It was, therefore, hypothesized that pre-resection SRS would improve the risk of LMD by sterilizing microscopic, dislodged tumor cells to prevent spread during surgery. [23, 24] In addition, another potential advantage of pre-operative SRS is a theoretical increased response to radiation due to intact vasculature and greater peri-tumoral oxygen content. [25–27] Studies have shown that lower doses of radiation are required for tumor control when the tumor has an intact blood supply and is oxygenated. This is due to radiation-induced DNA damage that ionizes oxygen molecules and generates oxygen-based free radicals which in turn damage nearby DNA resulting in tumor killing. [25, 26] In the setting of BMs, it is plausible that lower radiation doses are needed to control microscopic disease if the SRS is given prior to surgery. [26]

The first pre-operative SRS study was published by Asher et al where 47 patients were treated consecutively with neoadjuvant SRS before surgical resection, with 24 of those patients analyzed as part of a prospective trial, and the results showed 6-month and 12-month LC rate of 97.8 and 85.6% respectively (Table 3). [28] Subsequently Patel et al performed a multi-institutional retrospective comparison of outcomes and toxicities for pre-operative SRS compared to post-operative SRS for 180 patients. [29] The planning target volume (PTV) of pre-operative SRS had 0 mm margin expansion from gross tumor volume (GTV) compared to 2 mm margin for PTV of post-operative SRS. The study did not note any statistical difference in the rates of local recurrence, distant brain recurrence, and OS but the pre-operative SRS cohort demonstrated significantly lower rates of symptomatic RN (4.9% vs. 16.4%, *p* = 0.01) and LMD (3.2% vs. 16.6%, p = 0.01) than their post-operative SRS counterpart. [29] The abstract of an updated retrospective analysis of 117 patients with 125 lesions treated with pre-op SRS is now available online (https://www.redjournal.org/article/S0360-3016(18)31346-4/fulltext) and reported the 1 and 2-year cavity LC rate to be 80.1 and 74.9%, distant in-brain failure (DBF): 45.3 and 60.2%, and LMD: 4.3 and 4.3%, respectively. Nevertheless, the results were likely limited by some intrinsic selection bias, as the two retrospective cohort groups were statistically different in their baseline performance status (Figure 1).

**Table 3:** Published studies of Pre-op SRS prior to Metastases Resection

Study	Treatment Modalities (Number of patients)	Target	Median survival (months)	Outcomes			
				LR (%)	DBF (%)	LMD (%)	Symptomatic RN (%)
Asher, 2014 [28]	PreOP SRS (47)	GTV = PTV	NA	14.4 (1 yr)	38.2 (1 yr)	NA	NA
Patel, 2016 [29]	PreOP SRS (66) Vs. Postop SRS (114)	GTV = PTV PTV = cavity + 1–2 mm margin	17.1 13.5	15.9 (1 yr) 12.6 (1 yr)	32 (1 yr) 39.1(1 yr)	3.2 (1 yr) 3.2 (2 yr) 8.3 (1 yr) 16.6 (2 yr)	4.9 (2 yr) 16.4 (2 yr)
Patel, 2017 [44]	PreOP SRS (66) Vs. Postop WBRT [36]	GTV = PTV PTV = cavity + 1–2 mm margin	13.9 12.6	24.5 (2 yr) 25.1 (2 yr)	53.2 (2 yr) 45 (2 yr)	3.5 (2 yr) 9 (2 yr)	5.6 (2 yr) 0 (2 yr)
Prabhu, 2018 (Abstract only)	Pre-OP SRS (117)	GTV = PTV	17.2	19.9 (1 yr); 25.1 (2 yr)	45.3 (1 yr); 60.2 (2 yr)	4.3 (1 yr); 4.3 (2 yr)	2.6 (1 yr) 4.8 (2 yr)

In this prospective phase II trial, we will establish the efficacy of pre-operative SRS in local disease control. Since the tumor will be resected within 4 days of the delivery of the radiation, we will treat lesions up to 5 cm in size with SRS. In addition, there is no available published literature that describes early tumor cell molecular responses to radiosurgical doses of ionizing radiation in humans. We will simultaneously perform an exploratory analysis regarding histologic and molecular changes after radiosurgery for specimens removed to determine correlation between response to radiation at tumor margin and LC. This analysis will potentially provide molecular prognostic data for local and overall outcomes of patients with BMs.

### Study goals and objectives

#### Primary objective

To evaluate 6-month in-brain local control utilizing pre-operative SRS followed by surgical resection for up to 4 brain metastases.

#### Secondary objectives

- Overall survival
- Progression free survival
- Distant-in-brain failure
- Rate of leptomeningeal spread
- Rate of radiation necrosis.

#### Exploratory objectives

Molecular studies to investigate the relationship between radiation dose and DNA damage in tumor tissue. In addition, characterization of early histologic and molecular changes, in terms of gene expression, seen within the tumor following radiation.

## Methods/design

### Study design

This is a single-center, single-cohort, single-arm, prospective phase II trial to determine the local control at 6 months utilizing pre-operative SRS followed by surgery within 1–4 days of radiation treatment in neurologically symptomatic patients with up to 4 brain metastases.

### Study population

Patients with 1–4 metastatic lesions in the brain identified on a diagnostic brain MRI or CT with at least one meeting the criteria for surgical resection i.e. symptomatic or size > 3 cm, will be identified as potential study candidates. Amongst the study candidates, patients meeting the inclusion and exclusion criteria (Table 4) will be eligible to enroll in the study. Eligible patients who complete the Informed Consent Process will be registered in the OnCore® database and assigned a patient ID number for the clinical study.

**Table 4:** Inclusion and Exclusion Criteria

Inclusion Criteria	
1. Radiographically confirmed solid tumor brain metastases 2. Criteria for surgical resection of at least one metastasis per neurosurgeon discretion 3. A diagnostic MRI Brain or CT Head demonstrating the presence of 1–4 solid tumor brain metastases and lesion to be resected no more than 5 cm in any direction, performed within 30 days prior to stereotactic radiosurgery. If multiple lesions are present, then the total brain metastases volume can be no more than 30 cm^3^ excluding the lesion to be resected. 4. For known primary, ds-GPA estimated median survival no less than 6 months 5. For unknown primary, GPA estimated median survival no less than 6 months 6. Surgical candidate per neurosurgeon discretion 7. Surgical resection able to be performed within 1–4 days after radiosurgery 8. Stereotactic radiosurgery candidate per radiation oncologist 9. ≥ 18 years old at the time of informed consent 10. Ability to provide written informed consent and HIPAA authorization 11. Platelet count > 100 k/ml, Hgb > 7.5 g/dL, INR < 1.3, ANC > 1.5 k/ml 12. Patients currently on cytotoxic chemotherapy or immunotherapy are eligible, not including anti-VEGF therapy	
Exclusion Criteria	
1. Patients who received anti-VEGF therapy within 6 weeks prior to enrollment 2. Major medical illnesses or psychiatric impairments, which in the investigator’s opinion will prevent administration or completion of the protocol therapy and/or interfere with follow-up 3. Patients with more than 4 brain metastases on MRI Brain or CT Head 4. Lesion to be resected is more than 5 cm 5. Total volume of metastatic disease more than 30 cm3 excluding lesion to be resected 6. Patients with leptomeningeal metastases documented by MRI or CSF evaluation 7. Previous whole brain radiation therapy 8. Previous radiation therapy to lesion to be resected 9. Planned adjuvant focal therapy including additional radiation therapy to the brain 10. Not a surgical candidate per neurosurgeon’s discretion 11. Not a radiosurgical candidate per radiation oncologist’s discretion 12. Surgery unable to be performed between 1 and 3 days after radiosurgery 13. Women who are pregnant or nursing are not eligible as treatment involves unforeseeable risks to the fetus or child 14. Patients who have a known primary and have an estimated median survival less than 6 months per ds-GPA 15. Patients who have an unknown primary and have an estimated median survival less than 6 months per GPA	

### Trial status and project duration

The trial is scheduled to recruit 44 patients and is currently recruiting. The projected duration for the study is 3 years. The time line is outlined in Table 6.

### Stereotactic radiosurgery

SRS will be delivered utilizing gamma knife or linear accelerator-based techniques. With the Leksell Gamma Knife Perfexion®, Leksell GammaPlan® will be used to generate the treatment plan with respect to the head frame coordinate system created by localization. Target volume and isocenter determination will be based on a brain MRI with the patient’s head in the stereotactic frame. Linear accelerator based stereotactic localization will be performed using the Encompass® SRS thermoplastic mask immobilization system. The patient will undergo a 1 mm slice thickness helical CT scan that will be fused with the MRI brain T1-weighted post-contrast axial scan used for target delineation. The CT-MRI fusion maximum correlation error must be less than 1.5 mm.

The prescribed dose will be based on tumor diameter per RTOG 90–05 dosing criteria with the exception that the largest lesion diameter to be treated with 15 Gy will be 5 cm (Table 5). [27] Since it has been shown that brain metastases up to 4.5 cm can be safely treated with 15 Gy SRS either alone or with WBRT with no reported toxicity at 2–3 months following treatment. [30]

**Table 5:** Radiosurgery dose criteria

Maximum Tumor Diameter	Prescribed Dose
≤ 20 mm	24 Gy
21–30 mm	18 Gy
31–40 mm	15 Gy
40–50 mm	15 Gy

SRS will be delivered to each lesion that has not previously undergone treatment. Due to the volumetric summation constraint for the remaining metastases, no single, non-resected lesion greater than 4 cm will be allowed in the study. If any two lesions are within 0.8 to 2 cm of each other, the intervening midplane dose will not exceed 13 Gy. This may require treating each respective target with a lesser dose than dictated by the above dosing criteria to minimize toxicity. The dose to the critical structures, including optic pathway, brainstem, cochlea and medulla, must meet constraints as designated by Task Group 101. [31] If the above constraints cannot be met utilizing the prescribed radiosurgery dose, then the highest dose to the target volume will be used such that constraints can be met. This will be considered a minor deviation.

### Surgical resection

At least one of the 4 lesions has to be either larger than 3 cm or symptomatic to meet the surgical resection criteria. One to four days after radiosurgery, the dominant lesion(s) will be maximally resected and labeled tissue will be sent to the neuropathology department for clinical diagnosis and radiobiological correlative studies. If for safety concern or other considerations, gross total resection is not reached, the residual disease in the setting of subtotal resection will be closely observed given that it has been treated with a definitive dose of SRS, reserving salvage local therapy for cases of progression. [29] Patients who received subtotal resection will be recorded and analyzed as well for risk stratification.

### Correlative analysis

For the radiobiological studies, tissues will be examined for a) pathologic diagnosis b) immunohistochemistry for immune cell infiltrates and c) mitochondrial histochemistry to address DNA damage. In addition, to understand the effect of radiation dosing on tumor tissue at the molecular level, RNA and DNA sequencing will be performed on peripheral and central sites from the tumor specimen of each patient’s tumor samples. The early cellular gene expression changes in response to high dose radiation will be assessed. DNA sequencing and RNA expression will also be correlated with all clinical outcomes to establish prognostic molecular classifications. Protein analysis for apoptotic pathway activation will also be done on tumor cells to assess radiation induced tumor cell killing.

### Response and progression assessment in neuro-oncology brain metastases (RANO-BM) [32]

Following delivery of SRS and surgical resection, all patients will be assessed for their clinical performance status (GPA/ds-GPA/ ECOG performance status/KPS) as well as toxicity at follow-up intervals as detailed in Table 6. Serial MRIs will be analyzed per RANO-BM criteria for assessment of LC, DBF and cranial progression free survival. [32] Initiation or continuation of chemotherapy, immunotherapy or other systemic agents is allowed per medical oncologist discretion.

**Table 6:** Study Protocol and follow up timeline

	Screening (within 30 days of SRS)	Stereotactic radiosurgery	Surgery (1–4 days post SRS)	1 Month Follow Up^a^ (30 days post SRS)	Follow Up^b^ (every 3 m until 2 yrs)	Long Term Follow Up^c^ (> 2 yr. post SRS)
Radiation oncology consult	x					
Neurosurgical consult	x					
Medical History	x			x	x	x
Physical Examination	x			x	x	x
Vitals	x	x	x	x	x	x
ds-GPA/GPA/ECOG performance status/KPS	x			x	x	x
Diagnostic MRI Brain or CT Head	x					
MRI Brain Planning Scan^f^		x				
WBC, Hgb, platelets, INR, ANC^d^	x		x			
Urine pregnancy test	x					
Toxicity assessment				x	x	
MRI Brain^e^					x	x
Tissue collection			x			

^a^Variations of +/− 14 days from the scheduled visit are permitted

^b^Variations of +/− 30 days from the scheduled visit are permitted

^c^Subjects will be followed at physician’s discretion, approximately every 6 months after 2 years post SRS, per standard of care. Any MRI Brain, physical exam or vitals obtained at these appointments will gathered. However, if these procedures are not performed per standard of care, this will not be a deviation

^d^Repeating Hgb, platelets, INR and ANC at time of surgery is per discretion of neurosurgeon

^e^MRI Brain performed at Indiana University will have sequences including contrast, no contrast, FLAIR, DTI and PWI. If patient receives MRI Brain outside of Indiana University, a minimum of contrast, no contrast and FLAIR will need to be obtained and all sequences mentioned above are encouraged

^f^Variations of −30 days from the scheduled visit are permitted for linear accelerator based SRS, and may include the baseline screening MRI at the treating radiation oncologist’s discretion

### Leptomeningeal disease and radiation necrosis

Assessment for LMD and RN will be performed using contrast-enhanced MRI scans. LMD is defined as new subarachnoid, ventricular or parenchymal enhancing nodules, focal or diffuse pial enhancement, ependymal, sulcal, folia or cranial nerve enhancement. [33] RN is defined as a contrast-enhancing lesion with surrounding edema within previous radiation treatment fields. MR spectroscopy and perfusion weighted imaging (PWI) sequences will be analyzed to differentiate between RN and recurrent tumor. [34, 35]

### General monitoring

For patients presenting with signs and symptoms relatable to peri-tumoral edema, dexamethasone will be prescribed at a dose level per clinician judgment. For patients presenting with seizure, anti-seizure medication will be prescribed at a dose level per clinician judgment. No specific type of anti-seizure medication is recommended or prohibited. Subjects will be encouraged to remain in the study and maintain regular follow-up. Possible reasons for early withdrawal may include, but are not limited to, the following:

- Subject decides to withdraw from the study. This decision must be an “independent decision” that is documented in the source documentation
- The Principal Investigator and/or treating physician may choose to withdraw a subject from the study if there are safety or other concerns.
- Subject becomes pregnant.
- Subject non-compliance.
- Subject lost to follow-up.
- Subject death.

### Follow-up

Time-to-event analyses will be measured from the date of completion of radiation therapy (Day 0). As outlined in Table 6 patients will be followed at 1-month post radiosurgery, then at 3 month and every 3 months thereafter for 2 years. A detailed medical history, toxicity assessment and physical examination including vital signs will be performed at each visit. Each follow-up starting from 3 month follow up will also include a MRI with the following sequences: without contrast, with contrast, FLAIR, DTI and PWI. The MRI will be analyzed per RANO-BM criteria [32] for assessment of local control, distant in-brain failure and cranial progression free survival. The MRI will also be analyzed for radiation necrosis and leptomeningeal disease as discussed in methodology section. Cases concerning for radiation necrosis will be further analyzed using MR spectroscopy. After the 2 year follow up period, subjects will be followed according to their treating physician per standard of care approximately every 6 months). Patients will continue to be followed for survival information until death. All AEs considered related to trial medication will be followed until resolution, return to baseline, or deemed clinically insignificant, even if this occurs post-trial.

### Safety considerations

Investigators will conduct continuous review of data and patient safety. Monthly review meetings for moderate risk trials are required and will include the principal investigator, clinical research specialist and/or research nurse. Adverse events (AEs) will be recorded from the time of study intervention and for at least 30 days after treatment discontinuation, regardless of whether or not the event(s) are considered related to trial medications. Any death occurring within 30 days after the study intervention will be reported as a serious adverse event (SAE) regardless of attribution. AEs will be reported to the IRB within 5 days from becoming aware of the event if they are: [1] unexpected, [2] related or possibly related to study participation, and [3] suggests that the research places subject(s) or others at greater risk of harm than was previously known. SAE reports are entered into OnCore® monthly and reviewed by the Data Safety Monitoring Committee (DSMC) chair and/or coordinator monthly. Findings will be reported to the full DSMC at the time of study review. At any time during the trial, the study will be closed early if it is the opinion of the investigators that the risks (or benefits) to the patient warrant early closure of the study. Alternatively, the DSMC may initiate suspension or early closure of the study based on its review of the investigator reports.

### Data management

This study will utilize electronic Case Report Form completion in the OnCore® database. A calendar of events and required forms are available in OnCore®**.** The OnCore® database is a comprehensive, web-based, Clinical Trial Management System (CTMS) which utilizes an Oracle database. All documents will be kept according to applicable federal guidelines. Clinical trial data in OnCore® are periodically monitored by the IU Simon Cancer Center Data Safety Monitoring Committee.

### Quality assurance

Records of IRB review and approval of all documents pertaining to this study will be kept on file in the Clinical Trials Office and are subject to inspection at any time during the study. Periodic status reports will be submitted to the Institutional Review Board at least yearly, as well as notification of completion of the study and a final report within 3 months of study completion or termination. Accrual data will be entered into the IU Simon Cancer Center OnCore® system. The Protocol Progress Committee (PPC) reviews study accrual twice per year while the PPC coordinator reviews accrual quarterly.

### Project Management

Patient recruitment is based on the referral from neurosurgeons, medical oncologists, radiation oncologist, primary care physicians, or self-referral. The principal investigators are responsible for checking eligibility and explaining the study principles, including detailed experimental schedule, investigational treatment, potential risks, and benefits. Radiation oncologists, neurosurgeons, and medical oncologists will be responsible for the consults, clinical treatments and follow up evaluations for the respective specialties. Pathologists will be performing histochemistry, immunochemistry and assist on other aspect of correlative studies. Statistical analysis will be performed by statisticians from the Department of Biostatistics at Indiana University School of Medicine.

### Statistical analysis

A sample size of 44 patients will provide 82% power with an alpha = 0.05 to determine if the proportion of patients with local control at 6 months is <= 83% or > = 95%. For the primary objective of 6-month local control for pre-operative SRS followed by surgical resection of the brain metastases, the proportion of patients who have local control at 6 months will be calculated along with a 95% confidence interval. Testing the observed proportion against a baseline 6-month local control proportion of 83% at a one-sided alpha of 5% using a binomial test will be done. The proportion of patients who have in-brain distant failure, radiation necrosis, and leptomeningeal spread in the evaluated patients will be summarized and exact binomial 95% confidence intervals will be determined. Additionally, time to local failure, distant in-brain progression, and overall survival will be calculated and analyzed using Kaplan-Meier methods with the medians estimated with a 95% confidence interval. The probability rates will be provided for 6 months, 1 year, and 2 years. Finally, the tissue biomarkers of interest in the molecular study will be correlated with treated lesion location, in-brain local control, in-brain distant control, and overall survival using Cox proportional hazards regression. Time dependent ROC curves will be generated to assess predictive ability.

## Discussion

To date, most studies have evaluated the role of SRS post-operatively in patients with clear indications for surgical management of BMs. [13, 20] We believe that pre-operative SRS merits further investigation as this technique has several clinical and radiobiological advantages, including precise tumor definition, possible sterilization of the tumor margin prior to surgical resection leading to decreased microscopic spread of disease during surgery, removal of irradiated tissue, and a theoretical radiobiological advantage of intact vasculature optimizing oxygenation. Additionally, all prior SRS studies limit indication of SRS to lesion < 4 cm in size due to dose related toxicity. Since we will be resecting the lesion post SRS, we will include lesions up to 5 cm in size.

We expect that, compared with historic LC rate associated with post-operative SRS, pre-operative SRS will have a higher LC rate as well as decreased risk of LMD and symptomatic RN. In terms of radiobiological studies, we expect differential gene expression profile in the tissue from the center of the lesion, which receives 50% greater radiation dose compared to the periphery of the lesion. In addition, we expect to see higher percentage of immune cell infiltrate in the radiation treated lesions.

Our exploratory analysis regarding histologic and molecular changes after radiosurgery will determine if any correlation between immune response to radiation at tumor margin and local control exists. This may characterize a group of patients requiring additional therapy after pre-operative radiosurgery and surgical resection for maximal tumor control.

## Abbreviations

BM: Brain metastasis
DBF: Distant-in-brain failure
ECOG: Eastern Cooperative Oncology Group
GPA/ds-GPA: Graded prognostic assessment/Diagnosis-specific graded prognostic assessment
GTV: Gross target volume
KPS: Karnofsky Performance Status
LC: Local control
LMD: Leptomeningeal disease
LR: Local recurrence
OS: Overall survival
PTV: Planning target volume
RN: Radiation necrosis
SRS: Stereotactic radiosurgery
WBRT: Whole brain radiation therapy
